# The impact of platform corporate venture capital vs. traditional corporate venture capital on internet initial public offering underpricing: Evidence from Chinese-listed internet firms

**DOI:** 10.3389/fpsyg.2022.984733

**Published:** 2022-09-20

**Authors:** Feng Fu, Shuangying Chen

**Affiliations:** ^1^School of Business, Chengdu University of Technology, Chengdu, China; ^2^School of Management and Economics, University of Electronic Science and Technology of China, Chengdu, China

**Keywords:** platform CVC, traditional CVC, internet IPO underpricing, signaling theory, motivation perspective

## Abstract

**Purpose:**

Platform firms are playing an increasingly major role in venture investment. Based on the motivation perspective and signaling theory, this paper examines the effects of platform corporate venture capital (CVC) versus traditional CVC on Internet IPO underpricing.

**Design/methodology/approach:**

The sample consists of 117 Chinese Internet firms that went public between 2004 and 2019. Two-stage Heckman regression analysis was used to test several hypotheses.

**Findings:**

This paper finds that, compared to traditional CVC firms, platform CVC firms increase Internet IPO underpricing. In particular, with the contingency of strong prior performance or implementation of China’s “Internet plus” policy, platform CVC firms increase Internet IPO underpricing more than traditional CVC firms. With increasing Internet penetration, platform CVC firms will increase Internet IPO underpricing less than traditional CVC firms.

**Practical implications:**

As CVC firms differ in their key resources and motivations used to realize their strategic goals, IPO firms should formulate their resource acquisition strategies according to their resource needs and the contexts in which they operate.

**Originality/value:**

By identifying the differences between platform CVC and traditional CVC, this paper complements previous research on the role of CVC backing of IPOs and extends the knowledge of CVC investment by shedding light on the contingency value of corporate investors and Internet IPO underpricing in emerging markets.

## Introduction

Research suggests that the presence of corporate venture capital (CVC) is likely to significantly influence the initial public offering (IPO) underpricing (Wang and Wan, 2013; Qiao et al., 2017). By and large, most studies have found that CVC-funded IPOs tend to experience less underpricing than non-CVC-funded IPOs (Wang and Wan, 2013; Qiao et al., 2017), while some studies suggest that CVC firms might increase IPO firms’ underpricing, largely due to conflicts of interest between IPO firms and CVC corporate parents (Maula and Murray, 2001; Masulis and Nahata, 2009). These studies are primarily based on an implicit assumption of CVC firms’ characteristic homogeneity. In practice, however, different CVC firms are heterogeneous in their characteristics (Park and Steensma, 2012). According to their characteristics, CVC firms can be classified into various types, such as expertise-related and status-related CVC firms (Ginsberg et al., 2011), and strategic and financial CVC firms (Chesbrough, 2002; Ivanov and Xie, 2010). Thus, a few studies have evaluated the differing impacts of CVC type on IPO underpricing. As suggested by Ginsberg et al. (2011), considering different types of CVC firms can give a more nuanced understanding of the effects of CVC firms on IPO underpricing. To further complement extant research, this paper focuses on CVC types and examines the effects of different CVC investments on the underpricing of IPO firms.

In particular, a new and powerful type of CVC, platform CVC which refers to the VC companies owned by Internet platform firms, has recently played an increasingly active role in venture investment (Langley and Leyshon, 2017). For instance, Internet platform firms (hereafter, “platform firms”) such as Google and Baidu are always among the top ten most influential and active CVC programs (CB Insights, 2019). With the popularity of platform capital, some studies have started to examine the value-adding contributions made by platform investment to the commercial success of their portfolio firms (Ceccagnoli et al., 2012; Mukhopadhyay et al., 2016; Langley and Leyshon, 2017). They found that partnership with a dominant platform increases entrepreneurial firms’ likelihood of IPO success. However, how the presence of platform CVC influences firms’ IPO underpricing remains unknown.

Moreover, relative to traditional CVC which refers to the VC companies owned by non-Internet platform firms, platform CVC may have a variable ability to support IPO firms because their parent corporations are significantly different from traditional firms in terms of resource profiles and strategic development orientations. More specifically, most parent corporations of platform CVC are well-known platform owners in platform-based Internet industries, such as Alibaba and Tencent. These platform owners are viewed as digital infrastructures that enable two or more groups to interact for value co-creation (Srnicek, 2017b; Schreieck et al., 2019). They possess the key resources, such as the numbers of users, technological expertise and data (Gawer and Cusumano, 2014; Boudreau and Jeppesen, 2015; Tiwana, 2015; Mukhopadhyay et al., 2016), and have an expansionary nature (Srnicek, 2017a) as well as network effects (McIntyre and Srinivasan, 2017). Notably, platform firms are different from traditional firms in organizational structure and characteristics, and in the resources they provide to portfolio companies. Given the variety of corporate parents, platform and traditional CVC may have different effects on IPO underpricing. However, empirical evidence of this is rather limited.

Initial public offering underpricing refers to the difference between the IPO offer and closing prices on the first day of trading (Ritter, 1998; Heeley et al., 2007). This study compares the impacts of the platform and traditional CVC on IPO underpricing with consideration of the motivation view and signaling theory. Given that the motivations of different CVC firms vary according to the motivation view (Arthurs et al., 2008; Wang and Wan, 2013), the platform and traditional CVC firms may be associated with different decision-making in relation to IPO offer prices. Furthermore, based on signaling theory, the platform and traditional CVC firms should have different effects on IPO closing prices because their different resource profiles convey different information to the investment market about the sustainable future growth potential of IPO firms. Specifically, platform CVC firms are more capable of providing Internet-related resources, while traditional CVC firms have broader and potentially greater access to offline assets. Differences in the resources profiles of the two types of CVC firms create heterogeneity in ties and outcomes for their funded IPO firms, especially those in specific industries (Park and Steensma, 2012). Consequently, based on these two arguments, this paper posits that platform and traditional CVC firms are likely to have different impacts on IPO underpricing.

This argument is tested in the empirical context of Chinese-listed Internet firms, which suits this study for three reasons. First, Internet IPOs have recently attracted considerable attention from the media, VC firms, and investors. They are likely to experience greater underpricing compared to other firms (Loughran and Ritter, 2004). Second, in China, the Internet industry accounts for nearly 40% of CVC firms’ investment (Tian, 2018). Particularly, most Chinese platforms are engaged in a large number of CVC investments, which have been the major sources of investment in new Internet firms (Tian, 2018). Third, China has continuously strengthened its Internet infrastructure, fostered new growth models of the Internet economy, and promoted the development of “Internet Plus” as the government has pledged more facilitating measures.

Research indicates out that the contingent value of CVC funding needs to be further explored (Park and Steensma, 2012), particularly in developing contexts (Drover et al., 2017). Thus, the development of the Internet economy in China is beneficial for exploring more contingencies and discussing how these various levels of situational factors impact the relationship between CVC type and Internet IPO underpricing. Overall, this study will mainly address two research questions: (1) How do platform and traditional CVC firms influence Internet IPO underpricing differently? (2) How do factors at the firm level (i.e., prior performance), industry level (i.e., Internet penetration) and country level (i.e., “Internet Plus” policy) augment and constrain these relationships? The results show that platform CVC firms increase Internet IPO underpricing. Additionally, the results also indicate that, compared with traditional CVC, the increasing effect of platform CVC on Internet IPO underpricing can be strengthened by strong prior performance and the “Internet plus” policy, and restrained by a high level of Internet penetration.

These findings contribute to the literature in three major ways. First, by identifying the differences between platform CVC and traditional CVC, this paper complements previous research on the role of CVC backing of IPOs. Specifically, prior studies have tended to combine all CVC firms into one category and are primarily based on an implicit assumption of CVC homogeneity (Ginsberg et al., 2011; Sahaym et al., 2016; Hahn and Kang, 2017). The present study extend the insights by identifing the difference between platform and traditional CVC firms, finding that they have different effects on Internet IPO underpricing, which impacts the sustainable development of IPO firms differently. Furthermore, this study enriches the framework of CVC firms to explain its implications for Internet IPO underpricing based on the motivation view and signaling theory. More specifically, this paper emphasizes both the motivations and resource profiles of platform and traditional CVC firms in developing the arguments. This paper not only highlights platform and traditional CVC firms’ different levels of motivation to IPO underpricing, but also views the presence of them as different signals that affect investor enthusiasm. By considering both the offer price and closing price, this study helps to provide a more nuanced understanding of how the motivations and resource profiles associated with different CVC firms influence Internet IPO underpricing. Finally, the findings of this study extend CVC-backed IPO research by indicating the contingency value of CVC firms and Internet IPO underpricing in China, where the government tends to foster new growth models for the platform economy and is promoting the development of the “Internet Plus” policy.

The rest of this study proceeds as follows. Section 2 describes the related theoretical background and explains the hypotheses. Next, Section 3 describes the sample and empirical methods. Then, Section 4 provides descriptionstatistics and empirical results. Finally, Section 5 discusses the main findings, theoretical contributions, managerial implications, and suggestions for future research.

## Literature review and hypothesis development

### Literature review

#### Venture capital types and initial public offering underpricing

Underpricing is a common occurrence for firms’ IPOs; that is, IPO firms often sell their equity at an initial offer price that is less than the closing price determined by the stock market. The presence of VC firms is a crucial part of the IPO process and can decrease the offer price or increase the closing price of IPO firms (Certo et al., 2001; Park and Steensma, 2012; Wang and Wan, 2013). The motivation view emphasizes that different VC firms, such as CVC and independent venture capital (IVC) firms, which are the two most dominant and influential types, typically hold different attitudes to underpricing. It focuses on how underpricing is caused by different motivations for setting the offer price (Wang and Wan, 2013). For instance, most established firms operate CVC programs to achieve strategic benefits rather than purely financial returns, while IVC firms often seek financial goals and tend to take their ventures to IPO as quickly as possible. Thus, IVC firms prefer to further discount the offer price, which may result in higher levels of underpricing than with CVC-backed IPOs (Lee and Wahal, 2004; Chen et al., 2011).

Moreover, according to signaling theory, VC firms with resource endowment are always viewed as a signal of firm quality by first-day investors. They play a key role in impacting IPO underpricing through the closing price. Importantly, different types of VC firms with distinct resource profiles convey different signals to investors (Wang and Wan, 2013). For example, IVC firms are generally professional in helping funded IPO firms obtain financial resources, while CVC firms can supply more valuable complementary assets from their corporate investors, including expertise, distribution, marketing and customer service (Maula et al., 2003). Therefore, the presence of CVC firms sends a stronger signal to first-day investors about the quality and future value of an IPO firm than the presence of IVC firms. As signaling theory has emphasized (Brau and Fawcett, 2006), signals interpreted by first-day investors as positive may increase their willingness to pay a premium over the offer price to acquire equity in IPO firms (Certo et al., 2001). This scenario creates a higher closing price and can cause large differences between the closing and offer prices, which increases IPO underpricing.

Overall, research has primarily used a motivation view or signaling theory to study the effect of VC type on IPO offer or closing prices, respectively. According to the conceptualization of IPO, it is more reasonable to understand the IPO underpricing phenomenon by considering both decreases in offer prices and increases in closing prices in relation to a combination of the motivation view and signaling theory.

#### Corporate venture capital types: Platform and traditional

According to this phenomenon that Internet platform firms, which are distinct from non-Internet platform firms in terms of resource profiles and strategic development orientations (Chakravarty et al., 2014), are playing an increasingly major role in venture investment (Langley and Leyshon, 2017; CB Insights, 2019), platform CVC firms is defined as VC companies owned by Internet platform firms, and traditional CVC firms as VC companies supported by non-Internet platform firms.

Distinct from traditional relationships, a platform is a digital infrastructure that enables two or more groups to interact for value co-creation (Srnicek, 2017b; Schreieck et al., 2019). To continually strengthen their market position, platforms should connect and attract more users, and ensure that the “co-creation of value” between users flows through the platform (Langley and Leyshon, 2017). This process can benefit the platform by generating greater network effects (Evans and Gawer, 2016) and also reflect the salience of user orientation to enable the platform’s competitiveness. As the extant studies show, firms with more customers would lead to effective flows of inventory, information, and working capital (Zhang et al., 2015). These potential advantages benefit firms from reducing the credit risk in financial market (Liu et al., 2021).

In contrast, traditional firms have a different business model, where interaction between parties is not a necessary condition for value generation (Chakravarty et al., 2014). For example, Ford’s suppliers are not viewed as its customers or required to interact with Ford’s users on the buy-side. Thus, the platform has stronger dependent relationships with marketplace users than traditional firms (Chakravarty et al., 2014). The different degrees of dependence on users can impact the attitudes of platform and traditional CVC firms to the setting of offer prices.

Moreover, compared to traditional firms, platforms can provide specific complementary resources. These include user bases, technological expertise, and data, with which complementary parties can develop and commercialize their own products and services (Gawer, 2009; Boudreau and Jeppesen, 2015; Tiwana, 2015; Mukhopadhyay et al., 2016). Specifically, a user base is associated with Internet traffic, which creates network effects and constitutes an important intangible asset for platform firms that the stock market values over and above accounting summary measures (Trueman et al., 2000; Rajgopal et al., 2003). Moreover, dominant platform firms have become data hubs, which gives them a key advantage over traditional firms since data is the basic resource that drives firms and provides a competitive advantage (Srnicek, 2017a). Xu and Liu (2020) have demonstrated the significant impact of physical and financial resource on firm profitability, productivity and market value. Therefore, due to the specificity and necessity of these resources in the Internet economy, platform CVC firms can convey a more positive signal of IPO firms’ value to investors than traditional CVC firms.

In particular, Internet firms are likely to have a stronger dependence on and more specific interaction with platform firms than traditional firms. The success of Internet start-ups, in the first instance, depends on significant investment in the Internet technology and know-how necessary to design and operate an infrastructure (Langley and Leyshon, 2017), and the ability to rapidly and consistently acquire Internet users. Relationship-specific investment from a platform CVC firm can give Internet start-ups access to specific complementary resources. Some scholars have demonstrated that it is a viable and successful strategy for Internet start-ups to be bought-out by a dominant platform (Langley and Leyshon, 2017). However, unlike platform firms, traditional firms are less dynamic and more closed. It is hard for them to provide specific resources and allow different parties to participate (Schreieck et al., 2019). Thus, this paper assumes that platform and traditional CVC may have different effects on Internet IPO underpricing.

### Hypothesis development

Following prior studies on the effects of VC type on IPO underpricing, this study integrates the motivation view and signaling theory to develop hypotheses on how platform and traditional CVC firms, affect Internet IPO underpricing. The motivation view concentrates on whether platform and traditional CVC firms are motivated to play active roles in setting appropriate offer prices, which may reflect different attitudes to Internet IPO underpricing. Signaling theory predicts that CVC firms’ existing resources and capabilities serve as signals that influence outside investors’ confidence in Internet IPO firms. The theory predicts that platform CVC and traditional CVC will have different effects on the IPO underpricing of Internet firms, which acts through effects on closing pricing.

#### Initial public offering underpricing: Platform CVC vs. traditional CVC

As noted above, platforms have relatively strong dependent relationships with marketplace participants and, hence, are more motivated to focus on users’ benefits than traditional firms (Chakravarty et al., 2014). Dominant platform firms can provide specific complementary resources for Internet start-ups (Gawer, 2009; Boudreau and Jeppesen, 2015). Thus, this paper emphasizes that platform CVC has a more positive effect on Internet IPO underpricing than traditional CVC for the following reasons.

First, from the motivation view, the platform CVC firm prefers to discount the offer price to create greater awareness among users of Internet IPO underpricing. More specifically, firms are willing to tolerate underpricing because it creates a need or desire to obtain more awareness among mass users (DuCharme et al., 2001). As for platforms, their user bases are their key resource, as they have the capacity to cultivate and capture value (Hagiu and Hałaburda, 2014; Staykova and Damsgaard, 2015). A platform CVC firm whose parent corporation has stronger dependent relationships with users is more likely to attract more potential users by generating popular awareness in the stock market by IPO underpricing than other categories of CVC firms (DuCharme et al., 2001). Thus, the platform CVC firm tends to set the lower offer price. Conversely, traditional firms are often less dependent on users because of their different business model. That is, interactions between different sides are not necessary for traditional firms to generate value (Chakravarty et al., 2014). A traditional CVC may prefer to set an offer price accurately than to attract users’ attention. Therefore, this studies predict that there will be a lower offer price for Internet IPO firms backed by platform CVC than those backed by traditional CVC.

Second, according to signaling theory, platform CVC firms with specialized complementary resources give a more positive signal of IPO firm quality to investors and creates a higher closing price for Internet IPOs because specialized resource profiles are unavailable with traditional CVC firms. As subsidiaries of the dominant platform, a platform CVC firm is associated with the number of Internet users, advanced Internet technology, and masses of data on products and the market (Gawer, 2009; Boudreau and Jeppesen, 2015; Srnicek, 2017a). However, developing these specialized complementary assets internally is generally not feasible for new Internet firms due to prohibitive costs and the difficulties of developing such assets in the short-run (Staykova and Damsgaard, 2015). The support provided by a platform CVC firm not only reduces the cost of acquiring specialized complementary resources for Internet firms, but also enhances their ability to gain earnings which determine the long-term sustainability of firms (Durana et al., 2022a,b). As a result, compared to a traditional CVC firm, a platform CVC firm can provide a more positive signal of an Internet IPO firm’s current quality and to first-day investors. The investors are willing to pay more for the same stocks they could not obtain at the initial offer price, which eventually leads to a higher closing price and more IPO underpricing. From these views, the next hypothesis is proposed.

*H1*: Platform CVC firms are more positively related to Internet IPO underpricing than traditional CVC firms.

#### The contingency mechanisms

Research regarding the effect of CVC on IPO firms also focuses on contingent relationships (Dushnitsky and Lenox, 2005; Ivanov and Xie, 2010). Some studies suggest that the magnitude of the impact of CVC investment is likely to vary between IPO firms and industries. For instance, Park and Steensma (2012) used a bivariate probit model of 508 samples to find that the effect of CVC on IPO firms largely hinges on situational factors, such as the resource needs of IPO firms and the industry’s environmental uncertainty. Sosnowski (2022) investigated the ersistence of pre-IPO earnings in the context of Central and Eastern European capital markets, and emphasized that the analysis of IPOs need to take into account regional specifics. National development, such as business climate and institutional strength, is directly or indirectly relevant in explaining the IPO phenomena (Jamaani and Ahmed, 2022). Thus, it is necessary to identify more contingencies and discuss how various levels of these situational factors affect CVC firms’ investment activitiesparticularly in developing contexts (Drover et al., 2017). To advance this line of research, this paper further explores how a firm-level factor (i.e., prior performance), an industry-level factor (i.e., Internet penetration) and country level (i.e., “Internet Plus” policy) moderate the effect of CVC type on Internet IPO underpricing.

##### Moderating effect of prior performance

Initial public offering firms that obtain superior performance commonly possess stronger capabilities and resources (Zheng et al., 2015). Existing resources and capabilities can, in turn, potentially impact the strategic relationship between IPO firms and CVC investors (Brau and Fawcett, 2006). This paper argues that platform CVC will increase the underpricing of Internet IPO firms with strong prior performance more than traditional CVC will, for the following reasons.

Relative to weak prior performance, strong performance enhances the willingness of a platform CVC firm to discount the offer price. More specifically, strongly performing Internet firms have better capability to bear the cost of higher underpricing and can recover such losses in subsequent offerings (Chua, 2014). As such, strongly performing Internet firms are not significantly concerned with underpricing. They are more willing to accept IPO underpricing in exchange for certain advantages (Leitterstorf and Rau, 2014). Hence, there is a greater incentive for platform CVC firms to attract potential users for both themselves and strongly performing Internet IPOs by underpricing, and to tolerate the resulting lower offer price.

In addition, relative to weak prior performance, Internet IPO firms with strong performance backed by a platform CVC firm face more underpricing *via* a higher closing price. On the one hand, strongly performing firms are more likely to possess the capability to exploit additional resources to grow sales or profits. Hence, Internet IPO firms with strong prior performance can more effectively convert the specialized complementary resources provided by a platform CVC firm into future performance. On the other hand, strong performance increases the likelihood that the platform CVC firm will provide the specific resources that help new Internet firms develop rapidly. For these reasons, the involvement of platform CVC firms can be viewed by investors as a more positive signal of the future growth of IPO firms with strong performance. Investor enthusiasm helps increase market heat at the time of IPO and increases the closing price, thus leading to more underpricing. In contrast, traditional firms may have greater difficulty in providing specific complementary resources that improve investor confidence. Thus, Hypothesis 2 is proposed as follows.

*H2*: With strong prior performance, the positive relationship between platform CVC firms and Internet IPO underpricing is more strengthened than that of traditional CVC firms.

##### Moderating effect of internet penetration

Internet penetration is the percentage of a population that uses the Internet (Salmons, 2008). China’s Internet penetration was about 60% at the end of 2018, above the global average that year. China’s increasing Internet penetration reflects the development of its telecommunication network, IT infrastructure, and Internet user base (Jibril et al., 2020). This paper expects that with high Internet penetration, the positive relationship between platform CVC firms and Internet IPO underpricing can be more weakened than that of traditional CVC firms, for the following reasons.

First, relative to weak Internet penetration, high Internet penetration weakens a platform CVC firm’s motivation to discount the offer price. Specifically, high Internet penetration means widespread Internet access and information acquisition (Salmons, 2008), which makes it easier for investors and users to obtain meaningful and objective information about Internet IPO firms. Such information probably makes investment and consumption behavior more rational. It is hard for a platform CVC firm to attract more potential users’ attention by IPO underpricing. As a result, the platform will have a low willingness to discount the offer price and create consumer awareness *via* Internet IPO underpricing.

Second, compared to weak Internet penetration, high Internet penetration reduces the effect of the positive signal conveyed by the specialized complementary resources of platform CVC firms which, in turn, decrease the Internet IPO’s closing price. As some scholars have noted (Jibril et al., 2020), high Internet penetration means more developed Internet-related resources, such as telecommunication networks, IT infrastructures, and Internet user bases. Internet firms can obtain specialized Internet-related resources easily and cheaply from the open market. Hence, high Internet penetration reduces the cost and difficulty of developing specialized complementary resources for Internet IPO firms, which lowers their dependence on platform CVC firms. Accordingly, the positive signal received by investors about an Internet IPO firm being backed by platform CVC can be weakened. Based on the above arguments, Hypothesis 3 is proposed.

*H3*: With high Internet penetration, the positive relationship between platform CVC firms and Internet IPO underpricing is more weakened than that of traditional CVC firms.

##### Moderating effect of The “internet plus” policy

This paper also argues that the relationship between the two types of CVC and Internet IPO underpricing may be moderated by the “Internet Plus” policy. The “Internet Plus” policy refers to the application of the Internet and other information technologies in various industries to foster their development in China (State Council, 2015). The gist is to use the Internet as a crosscutting lever for integration with other areas of restructuring and to facilitate a new form of digital capitalism capable of uplifting the Chinese economy in the global setting (Liu, 2019). Notably, the “Internet Plus” policy is a vital influence on firms’ investment behavior and stock market heat. This paper expects that the “Internet Plus” policy will strengthen the positive effect of platform CVC on Internet IPO underpricing more so than traditional CVC, for the following reasons.

First, under the “Internet Plus” policy, platform CVC firms will have a stronger motivation for reducing the offer price for an Internet IPO firm. Specifically, the Chinese government encourages dominant platform firms with advanced internet technology to participate in the “Internet Plus” strategy. According to the institutional view (Kevin Zheng et al., 2017), platform firms have an important role in the “Internet Plus” plan and must respond to the government’s call to foster more successful new firms. Institutional pressure forces the platform firms to enhance their empowerment ability by accumulating more user resources. Thus, under the “Internet Plus” policy, a platform CVC firm will have stronger willingness to attract more potential users by underpricing than a traditional CVC firm and, simultaneously, ensure the Internet IPO success of their funded firms.

Second, the “Internet Plus” policy also increases the closing price of platform CVC-backed Internet IPOs. Under this policy, platform firms can get more government support than traditional firms. From the institutional view, such government support can give the platform CVC firm greater access to important Internet-related resources and the privileged incentives that foster new industries and business development. All these factors send positive signals about the future value of platform CVC-backed Internet IPO firms. Hence, the “Internet Plus” policy will enhance investor enthusiasm for platform CVC-backed Internet IPO firms, leading to higher closing prices. A higher closing price increases the likelihood of underpricing. Thus, Hypothesis 4 is proposed as follows.

*H4*: Under the “Internet Plus” policy, there is a stronger positive relationship between platform CVC firms and Internet IPO underpricing than with traditional CVC firms.

## Methodology

### Sample selection

The sample for this study was drawn from Chinese Internet firms that went public between 2004 and 2019. Considering that the number of Chinese Internet firms listed in some overseas markets is low, this paper collected data on the following markets: the Shanghai and Shenzhen Stock Exchanges, the main board in Hong Kong, and the NASDAQ in the United States. The initial sample consists of 135 Internet IPO firms.

To construct the database, venture capital data was collected from IPO prospectuses. Consistent with Ivanov and Xie (2010), this paper required that at least one VC firm that provided funding in the past to be listed as a shareholder in the IPO prospectus, since VC firms are unlikely to impact IPO performance if they have terminated their involvement with the IPO firm before the IPO occurs. Furthermore, the venture capital data is supplemented with the CVSource database. This contains detailed information regarding the characteristics of Chinese firms and their venture capital investors, such as exit routes (IPO vs. acquisition), exit dates, and types of venture capital funds (CVC vs. IVC). In addition, this paper used COMPUSTAT, WIND, and China Internet Network Information Center (CNNIC) databases to collect financial data, stock market information, and other control variables. After merging these data resources and excluding those with missing information, this paper obtained a final sample of 117 firm-year observations for analysis. The final sample was pooled at the firm level.

### Measures

#### Dependent variables

##### Initial public offering underpricing

Initial public offering underpricing represents that the issue price of IPO firm shares was less than the closing price at of their first day of trading, and reflects a positive first-day return (Aggarwal et al., 2002; Ragozzino and Reuer, 2011). Following prior work in financial economics (Wang and Wan, 2013; Chen et al., 2015; Qiao et al., 2017), this paper calculated *IPO underpricing* as the first-day closing price minus the offer price, divided by the offer price, using the following formula:

IPO underpricing = (Price at close of 1st trading day − Offer price)/Offer price.

#### Explanatory variables

##### Platform CVC

An IPO firm was identified as backed by Internet platform corporate investor if any of the following organizations were among its shareholders: other public Internet platform firms, VC companies owned by other Internet platform companies, or companies with some Internet platform companies as major shareholders. The platform CVC indicator variable was assumed to have a value of 1 if an IPO firm received funding from at least one Internet platform corporate investor and 0 otherwise.

##### Traditional CVC

The traditional CVC took a value of 1 if a new public firm received funding from a non-Internet platform corporate investor and 0 otherwise. Consistent with prior studies (Park and Steensma, 2012), some platform CVC-funded IPO firms were also funded by traditional CVC. Thus, traditional CVC constituted a control group used to assess the value of platform CVC for IPO firms.

#### Moderator variables

##### Prior performance

Sales growth best reflects the current resources and capabilities relevant to firms’ success (Zheng et al., 2015). Hence, this paper used prior sales growth as the indicator of prior performance, which is measured as the percentage change in Internet IPOs’ sales revenue each year: (Salest – Salest − 1)/Salest − 1, where *t* represents the year. Prior sales growth is a consistent, current and measurable indicator of capabilities, which captures the product and quality differences across firms that may have affected market investors’ reactions.

##### Internet penetration

This paper used annual data of Internet penetration rate published by CNNIC to measure this variable. Internet penetration rate corresponds to the percentage of the total population of a country or region that uses the Internet (Salmons, 2008), and also reflects the availability and usage level of network infrastructure.

##### Internet plus policy

The State Council issued The Guidance of the State Council on Actively Promoting the Internet Plus Action on July 4, 2015. Considering the emergence of the policy’s effects, this paper coded this variable 1 if the platform firm went public after 2015 or 0 otherwise.

#### Control variables

To control for possible additional factors that influence IPO underpricing, this paper used the following variables. Firm age was used as an indicator of general firm quality and was calculated as the number of years between a firm’s founding and its IPO date. Firm scale was calculated as the firm’s number of employees. Larger firms may make greater CVC investment because they tend to have greater resources and abilities to make discretionary or uncertain investments. Leverage is the degree to which the firm was leveraged, and was expressed as the ratio of its debt to assets. Since governance parameters can serve as useful screening and sorting criteria and can affect investors’ valuation of IPO firms, this paper also controlled for Board size using the total number of directors on the board. Additionally, IVC was a dummy variable set to 1 if the IPO was backed by independent venture capital or 0 otherwise. Location was coded 1 if the firm was public in the Shanghai and Shenzhen Stock Exchanges or 0 otherwise.

This paper also controlled for additional CEO background-related variables that may influence venture capitalist selection and firm IPO underpricing. Founder was defined as 1 if the CEO was a founder of the firm or 0 otherwise. Entrepreneur refers to the CEO’s entrepreneurial experience and was 1 if the CEO had founded at least one business or 0 otherwise. Work experience was measured as 1 if the CEO had worked in at least two different firms or 0 otherwise. CEO share was measured as the share percentage owned by the CEO.

### Analytical approach

Data of this study was structured as cross-sectional data because this study focuses mainly on the effect of CVC type on Internet IPO underpricing. Recent studies have emphasized the importance of correcting for selection bias and endogeneity because managers do not make strategic organizational decisions randomly, but based on expectations of how their choices affect firm performance (Kim et al., 2015). Hence, this paper employed a two-stage Heckman estimation procedure to examine the effect of platform CVC on Internet IPO underpricing. This paper took the above control variables and chose two additional variables (CEO Internet experience and Industry level of platform CVC) as the basic characteristic variables that can potentially correct for endogeneity. This paper estimated the first-stage equation as an independent probit model to predict whether or not firms had pursued platform CVC. Then, in the second stage regression, this paper included the inverse Mills ratio generated in the first-stage probit regression to adjust for potential selection bias. As Table 1 shows, the inverse Mills ratios were not significant, suggesting this study does not suffer from serious endogeneity problems.

**Table 1:** Regression results.

Variables	Model 1	Model 2	Model 3	Model 4	Model 5	Model 6
Platform CVC		0.151^*^(0.087)	2.299^*^(1.255)	0.522^**^(0.122)	2.276^*^(1.199)	2.366^**^(1.168)
Traditional CVC		0.007 (0.085)	2.043 (2.080)	0.027 (0.117)	1.880 (2.106)	2.375 (2.061)
Platform CVC ^*^Prior performance			2.406^*^(1.405)			2.491^*^(1.289)
Traditional CVC ^*^Prior performance			2.263 (2.304)			2.432 (2.248)
Platform CVC ^*^Internet penetration				−0.538^***^(0.141)		−1.765^***^(0.491)
Traditional CVC ^*^Internet penetration				0.188^*^(0.110)		0.260^**^(0.111)
Platform CVC ^*^Internet plus					1.363^***^(0.381)	1.306^***^(0.372)
Traditional CVC ^*^Internet plus					0.123 (0.179)	−0.280 (0.245)
Prior performance		0.013 (0.014)	−0.004 (0.017)	0.026 (0.020)	−0.008 (0.017)	−0.008 (0.016)
Internet penetration		−0.014^**^(0.006)	−0.013^**^(0.006)	−0.022^***^(0.006)	0.016 (0.036)	0.016 (0.035)
Internet plus		0.479 (1.841)	0.683 (1.854)	0.762 (1.858)	−1.416 (1.861)	−1.661 (1.814)
Inverse Mills ratio		−0.031 (0.081)	−0.025 (0.080)	−0.128 (0.099)	−1.416 (1.861)	−0.023 (0.074)
Firm age	0.002 (0.009)	0.001 (0.009)	0.001 (0.009)	−0.003 (0.011)	0.008 (0.009)	0.008 (0.008)
Founder	−0.177^*^(0.089)	−0.172^*^(0.090)	−0.151^*^(0.090)	−0.040 (0.114)	−0.094 (0.088)	−0.084 (0.086)
Board scale	−0.028 (0.019)	−0.029 (0.020)	−0.031 (0.020)	0.005 (0.027)	−0.029 (0.019)	−0.026 (0.018)
Firm scale	−0.009 (0.034)	−0.011 (0.038)	−0.006 (0.037)	−0.060 (0.050)	0.012 (0.034)	0.006 (0.033)
IVC	0.121 (0.085)	0.120 (0.085)	0.130 (0.085)	−0.043 (0.116)	0.084 (0.083)	0.077 (0.080)
Firm leverage	−0.004 (0.039)	−0.005 (0.040)	−0.007 (0.039)	−0.022 (0.055)	−0.010 (0.037)	−0.001 (0.036)
Work experience	0.079 (0.076)	0.042 (0.082)	0.061 (0.082)	−0.067 (0.107)	0.094 (0.076)	0.089 (0.074)
Entrepreneur	−0.047 (0.073)	−0.064 (0.074)	−0.089 (0.075)	−0.086 (0.102)	−0.133^*^(0.073)	−0.147^**^(0.072)
CEO_share	0.001 (0.003)	0.001 (0.003)	0.001 (0.003)	0.003 (0.004)	−0.002 (0.003)	−0.002 (0.003)
Location	0.339^**^(0.132)	0.347^**^(0.138)	0.316^**^(0.139)	0.508^***^(0.173)	0.263^*^(0.144)	0.235^*^(0.141)
_cons	1.025^**^(0.459)	1.053^*^(0.532)	1.012^*^(0.530)	1.735^***^(0.631)	0.677 (0.520)	0.942^*^(0.518)
Years	Yes	Yes	Yes	Yes	Yes	Yes
R-squared	0.682	0.686	0.690	0.693	0.725	0.739

*** *p* < 0.01;

** *p* < 0.05;

* *p* < 0.1; *N* = 117.

## Results

Table 2 reports the means, standard deviations, and correlations of the variables. A re of correlations among independent variables suggests that multicollinearity is not a major concern, as confirmed by the variance of inflation factor which did not exceed the generally accepted threshold of 10 (Cohen et al., 2014).

**Table 2:** Descriptive statistics and correlation.

Variables	Mean	SD	1	2	3	4	5	6	7	8	9	10	11	12	13	14	15	16
1. IPO underpricing	0.269	0.536																
2. CVC involvement	0.500	0.502	−0.031															
3. Platform CVC	0.300	0.460	−0.018	0.455^***^														
4. Traditional CVC	0.192	0.396	0.003	0.488^***^	−0.319^***^													
5. Firm age	8.138	5.237	0.059	−0.001	−0.117	0.159^*^												
6. Founder	0.646	0.480	−0.020	0.064	−0.042	0.116	0.078											
7. Board scale	7.962	1.914	−0.081	0.093	0.057	0.061	0.250^***^	−0.133										
8. Firm scale	7.200	1.481	−0.077	0.246^***^	0.315^***^	−0.022	0.317^***^	0.176^**^	0.278^***^									
9. IVC	0.808	0.396	−0.044	0.176^**^	0.106	0.090	−0.024	0.292^***^	−0.041	0.190^**^								
10. Firm leverage	0.681	0.863	−0.163^*^	0.128	0.168^*^	−0.031	−0.194^**^	0.262^***^	0.069	0.087	0.165^*^							
11. Location	0.269	0.445	0.253^***^	−0.156^*^	−0.360^***^	0.232^***^	0.459^***^	0.014	0.221^**^	−0.147^*^	−0.100	−0.269^***^						
12. Entrepreneur	0.400	0.492	−0.060	−0.031	−0.089	0.080	−0.070	0.440^***^	−0.124	0.019	0.199^**^	0.090	0.035					
13. CEO_share	21.389	19.737	−0.020	−0.091	−0.207^**^	0.100	0.124	0.541^***^	−0.069	0.109	0.137	0.133	0.154^*^	0.442^***^				
14. Work experience	0.738	0.441	−0.027	0.035	0.046	−0.021	−0.199^**^	−0.038	−0.012	−0.169^*^	−0.068	0.031	−0.073	−0.014	0.008			
15. Prior performance	1.263	2.270	−0.037	−0.038	0.026	−0.070	−0.274^***^	0.042	−0.208^**^	−0.083	0.052	−0.006	−0.268^***^	0.131	−0.018	0.113		
16. Penetration	44.892	14.479	−0.458^***^	0.203^**^	0.305^***^	−0.112	−0.083	−0.013	0.090	0.061	0.031	0.140	−0.199^**^	0.026	−0.011	0.171^*^	0.107	
17. Internet plus	0.515	0.502	−0.227^**^	0.200^**^	0.265^***^	−0.074	−0.081	0.055	0.037	0.080	0.035	0.084	−0.244^***^	−0.025	0.059	0.123	0.141	0.558^**^

*** *p* < 0.01;

** *p* < 0.05;

* *p* < 0.1.

Table 1 reports the estimates of the Internet IPO underpricing models. Model 1 included control variables. Model 2 tested the effects of platform CVC firms and traditional CVC firms on Internet IPO underpricing. The results show that platform CVC positively influences Internet IPO underpricing (*b* = 0.1151, *p* < 0.1), while traditional CVC does not (*b* = 0.007, *p* > 0.1), which supports Hypothesis 1. Furthermore, Model 3–6 presents the contingency mechanism for the effects of CVC type on Internet IPO underpricing. In Models 3–5, platform CVC and traditional CVC’s interactions with prior performance, Internet penetration and Internet plus were, respectively, added. Finally, model 6 included all interaction effects simultaneously. Results did not change significantly across different model specifications, which suggested that the findings were quite robust. Hence, this study tested hypotheses on the basis of the results of model 8, the most complete model specification.

**Figure 1:**
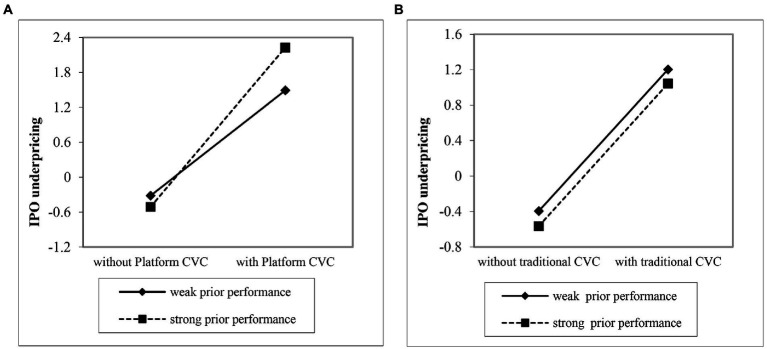
The moderating effect of prior performance. **(A)** Platform CVC. **(B)** Traditional CVC.

Hypothesis 2 stated that with strong prior performance by Internet firms, platform CVC firms will increase Internet IPO underpricing more than traditional CVC firms. In model 6, the interaction of platform CVC and prior performance is positive and significant (*b* = 2.491, *p* < 0.10); however, the interaction of traditional CVC and prior performance is not significant (*b* = 2.432, *p* > 0.10). These results support Hypothesis 2. To further probe this finding, this paper plotted the results in Figure 1. As is shown in Figure 1A, the slope of platform CVC and IPO underpricing is steeper with strong prior performance than with low prior performance. Meanwhile, Figure 1B shows that the slopes of the effect of traditional CVC on IPO underpricing for both strong and low prior performance are roughly parallel, which is consistent with Hypothesis 2.

**Figure 2:**
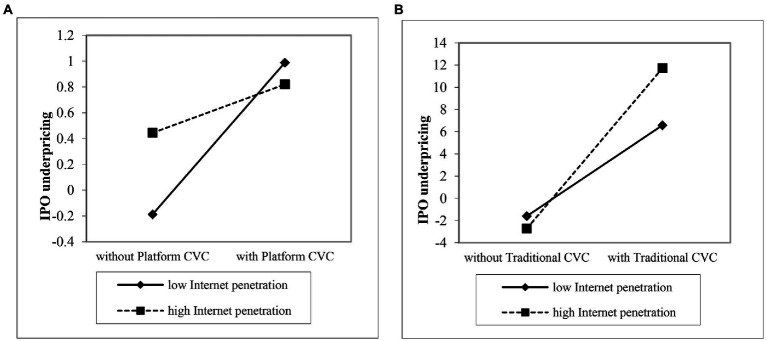
the moderating effect of Internet penetration. **(A)** Platform CVC. **(B)** Traditional CVC.

Hypothesis 3 proposed that with the increasing Internet penetration, the positive effect of platform CVC firms on Internet IPO underpricing is more weakened than that of traditional CVC firms. As the model 6 shows, the interaction coefficient of platform CVC and Internet penetration is negative and significant (*b* = −1.765, *p* < 0.01), yet the interaction of traditional CVC and Internet penetration is positive and significant (*b* = 0.260, *p* < 0.05). Hence, Hypothesis 3 is also supported. This significant moderating effect is plotted in Figure 2. As is shown in Figure 2A, the slope of platform CVC and IPO underpricing is more even with high Internet penetration than with low Internet penetration. Figure 2B shows that the slope of traditional CVC and IPO underpricing is steeper with high Internet penetration than with low Internet penetration, indicating that Internet penetration only weakens the positive effect of platform CVC on IPO underpricing.

**Figure 3:**
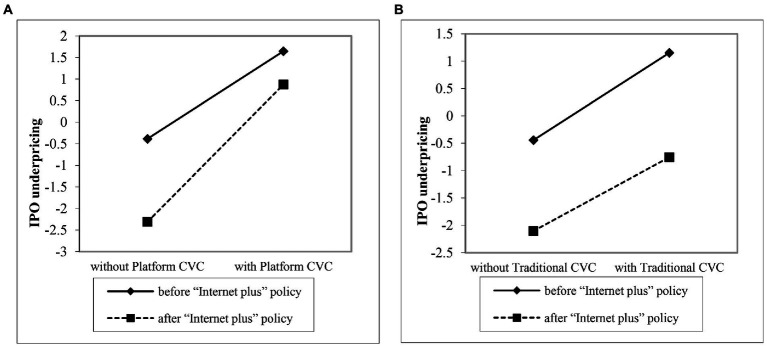
the moderating effect of “Internet plus” policy. **(A)** Platform CVC. **(B)** Traditional CVC.

Hypothesis 3 proposed that under the “Internet Plus” policy, platform CVC firms increases Internet IPO underpricing more than traditional CVC firms. The results of Model 6 show that the interaction of platform CVC and the “Internet plus” policy is positive and significant (*b* = 1.306, *p* < 0.01), and the interaction of traditional CVC and “Internet plus” is negative but not significant (*b* = −0.280, *p* > 0.10). Accordingly, Hypothesis 4 is supported. To further probe this finding, this paper plotted the results in Figure 3. As is shown in Figure 3A, the slope of platform CVC and IPO underpricing is steeper under the “Internet Plus” policy. However, Figure 3B shows that, before and after “Internet Plus” policy implementation, the slopes of the effect of traditional CVC on IPO underpricing are parallel, which provides further support for Hypothesis 4.

## Discussion

### General conclusions and implications

To summarize, using the sample of Chinese-listed Internet firms, this paper studied the influence of platform CVC and traditional CVC on Internet IPO underpricing. The results demonstrate that, platform CVC firms increases Internet IPO underpricing more than traditional CVC firms. In particular, when prior performance is strong, or the “Internet Plus” policy is in effect, the positive relationship between platform CVC firms and Internet IPO underpricing is more strengthened than that of traditional CVC firms. Additionally, with high Internet penetration, the positive relationship between platform CVC firms and Internet IPO underpricing is more weakened than that of traditional CVC firms.

### Theoretical implications

Theoretically, these findings contribute to the existing literature in three major ways. First, this paper have extended CVC-backed IPO research and demonstrated the importance of CVC type (platform or traditional), which is an under-researched dimension. Analysis of the relative effects of platform and traditional CVC is relevant in the empirical context of this study because of the active engagement of platform firms in new firms and their heavy investment in VC programs. Thus, unlike the prior studies which have tended to combine all CVC firms into one category and are primarily based on an implicit assumption of CVC homogeneity (Ginsberg et al., 2011; Sahaym et al., 2016; Hahn and Kang, 2017), the present study identified the difference between platform and traditional CVC firms, finding that they have different effects on Internet IPO underpricing. This result is consistent with studies of Ginsberg et al. (2011), Ivanov and Xie (2010) and Chesbrough (2002), they have shown that CVC type should be considered especially sophisticated due to their corporation parents’ diverse expertise and resources. Expanding their logic, this paper further identifies the differences between platform CVC and traditional CVC, and complements empirical research on the role of CVC backing in IPOs.

Second, this study extends the framework of CVC firms to explain its implications for Internet IPO underpricing based on the motivation view and signaling theory. More specifically, this paper emphasizes both the motivations and resource profiles of platform and traditional CVC firms in developing the arguments. This study not only highlight platform and traditional CVC firms’ different levels of motivation to IPO underpricing, but also view the presence of them as different signals that affect investor enthusiasm. This paper extends the prior work of Wang and Wan (2013), who propose that private and corporate VC have different inclinations toward IPO underpricing due to their different motivations and resource profiles. By considering both the offer price and closing price, this study helps to provide a more nuanced understanding of how CVC type relates to Internet IPO underpricing. In addition, these findings echo the study of Park and Steensma (2012), who showed that CVC funding is particularly beneficial for new firms that require specialized complementary assets at the time of IPO. Furthermore, this study extends their research by confirming that CVC firms are heterogeneous, and suggesting that CVC firms with specialized complementary resources can convey more positive information in stock markets for funded Internet IPO firms, which further enriches the signaling theory of venture capitalists.

Third, this study also contributes to the literature by shedding light on the contingency value of the link between CVC type and Internet IPO underpricing. Existing studies emphasize that the conditions under which IPO firms operate, and their capabilities, may influence the extent to which they benefit from CVC funding (Park and Steensma, 2012). However, there is little knowledge regarding the contingency value of CVC firms on firms’ IPO underpricing. Accordingly, this study demonstrates a contingency mechanism within the impacts of platform and traditional CVC in China, where the government tends to foster new growth models for the platform economy and is promoting the development of the “Internet Plus” policy. These findings show that, compared with traditional CVC, the increasing effect of platform CVC on Internet IPO underpricing can be strengthened by strong prior performance and the “Internet plus” policy, and restrained by a high level of Internet penetration. These results supports the studies of Sosnowski (2022) and Jamaani and Ahmed (2022), who proposed that the national development, such as business climate and institutional strength, is directly or indirectly relevant in explaining the IPO phenomena. As such, this study contributes to CVC-backed IPO research by indicating the contingency value of CVC firms and Internet IPO underpricing in emerging market.

### Managerial implications

The managerial implications of this study are straightforward. CVC firms differ in their key resources and motivations used to realize their strategic goals. Accordingly, the effect of CVC type on IPO firm valuation is variable. IPO firms should formulate their resource acquisition strategies according to their resource needs and the contexts in which they operate. Although a platform can supply specific complementary resources, platform CVC tends to have a stronger effect on IPO underpricing than traditional CVC. Given this, new firms can accept venture capital selectively according to their strategic needs.

### Limitations and future research directions

The study has limitations that provide avenues for further research. Firstly, the sample focused on Chinese-listed Internet firms. However, other non-Internet industries, such as the traditional manufacturing industry, may also be funded by platform and traditional CVC firms. Future research could explore their impacts on non-Internet firm IPO underpricing. Moreover, the research context is China, which has the largest Internet user base. Although China shares many features with other markets with rapidly growing platform economies, it also possesses its own unique institutional characteristics. Therefore, future studies are encouraged to test the generalizability of the propositions to different samples and economies.

Finally, like most CVC-backed IPO research, this study relies on secondary data rather than on data obtained from direct surveys of firm behavior. Future studies that use primary data (e.g., obtained through surveys and interviews) will contribute to a deeper understanding of the influence of platform CVC and traditional CVC on new ventures in different contingencies.

## Data availability statement

The original contributions presented in the study are included in the article/supplementary material, further inquiries can be directed to the corresponding author.

## Author contributions

FF processed the data and wrote the paper. SC designed and performed the research. All authors contributed to the article and approved the submitted version.

## Funding

This research was funded by the National Natural Science Foundation of China (71672020 and 72072020).

## Conflict of interest

The authors declare that the research was conducted in the absence of any commercial or financial relationships that could be construed as a potential conflict of interest.

## Publisher’s note

All claims expressed in this article are solely those of the authors and do not necessarily represent those of their affiliated organizations, or those of the publisher, the editors and the reviewers. Any product that may be evaluated in this article, or claim that may be made by its manufacturer, is not guaranteed or endorsed by the publisher.
